# Inflammatory Micro-Environmental Cues of Human Atherothrombotic Arteries Confer to Vascular Smooth Muscle Cells the Capacity to Trigger Lymphoid Neogenesis

**DOI:** 10.1371/journal.pone.0116295

**Published:** 2014-12-30

**Authors:** Kevin Guedj, Jamila Khallou-Laschet, Marc Clement, Marion Morvan, Sandrine Delbosc, Anh-Thu Gaston, Francesco Andreata, Yves Castier, Catherine Deschildre, Jean-Baptiste Michel, Giuseppina Caligiuri, Antonino Nicoletti

**Affiliations:** 1 Unité 1148, Institut National de la Santé et de la Recherche Médicale (INSERM), Hôpital Xavier Bichat, Paris, France; 2 Université Denis Diderot, Paris VII, Paris, France; Virginia Polytechnic Institute and State University, United States of America

## Abstract

**Background:**

Experimental atherosclerosis is characterized by the formation of tertiary lymphoid structures (TLOs) within the adventitial layer, which involves the chemokine-expressing aortic smooth muscle cells (SMCs). TLOs have also been described around human atherothrombotic arteries but the mechanisms of their formation remain poorly investigated. Herein, we tested whether human vascular SMCs play the role of chemokine-expressing cells that would trigger the formation of TLOs in atherothrombotic arteries.

**Results:**

We first characterized, by flow cytometry and immunofluorescence analysis, the prevalence and cell composition of TLOs in human abdominal aneurysms of the aorta (AAAs), an evolutive form of atherothrombosis. Chemotaxis experiments revealed that the conditioned medium from AAA tissues recruited significantly more B and T lymphocytes than the conditioned medium from control (N-AAA) tissues. This was associated with an increase in the concentration of CXCL13, CXCL16, CCL19, CCL20, and CCL21 chemokines in the conditioned medium from AAA tissues. Immunofluorescence analysis of AAA cryosections revealed that α-SMA-positive SMCs were the main contributors to the chemokine production. These results were confirmed by RT-qPCR assays where we found that primary vascular SMCs from AAA tissues expressed significantly more chemokines than SMCs from N-AAA. Finally, *in vitro* experiments demonstrated that the inflammatory cytokines found to be increased in the conditioned medium from AAA were able to trigger the production of chemokines by primary SMCs.

**Conclusion:**

Together, these results suggest that human vascular SMCs in atherothrombotic arteries, in response to inflammatory signals, are converted into chemokine-expressing cells that trigger the recruitment of immune cells and the formation of aortic TLOs.

## Introduction

Atherosclerosis is characterized by a chronic inflammatory process during which both innate and adaptive immune effectors play a role [1]. The canonical paradigm postulates that metabolic disturbances elicit a chronic, pathogenic inflammatory process in the intima of atherosclerotic arteries. From a mechanistic point of view, intimal inflammation does not provide a completely satisfactory framework to understand the involvement of adaptive immune cells. Indeed, besides the fact that leukocyte diapedesis across the arterial endothelium in early lesions must be a rare event due to the rapid flow conditions, once extravasated, immune cells might not be in an optimal micro-environment for their maturation and activation. Actually, the maturation and the induction of adaptive immune effector cells – notably that of B cells – require exquisitely regulated conditions that are ideally met in secondary lymphoid organs (SLOs), but not in the arterial intima. Interestingly, intra-tissue ectopic lymphoid structures that support adaptive immune response induction and maturation have been reported in tissues subjected to chronic inflammation [2]. An increasing number of studies have highlighted the formation of organized ectopic lymphoid structures in the adventitia of human and mouse atherosclerotic aortas [3]–[5]. These aortic tertiary lymphoid organs (TLOs) are composed of B cell follicles that resemble the ones in SLOs [6]. Their localization in front of the intimal atherosclerotic lesions may allow them to perceive and mount immune responses against plaque antigens radially convected towards the adventitia [7]. Immune effectors generated within these structures could hence be self-reactive and possibly participate to arterial tissue destruction. Deciphering mechanisms of TLO formation could therefore provide tools to interfere with the generation of local pathological immune effectors.

Based on our observations in the setting of human chronic rejection [8], we hypothesize that the program triggered during lymphoid neogenesis recapitulates the developmental program of SLO organogenesis. SLO developmental steps have been decrypted through genetic studies in mice. At the first place, a subset of hematopoietic cells, so-called “lymphoid tissue inducer (LTi) cells”, interact with stromal lymphoid tissue organizer (LTo) cells through α4β1/VCAM-1, lymphotoxin (LT)-α1β2/LTβR and tumor-necrosis factor (TNF)-α/TNFR. In response to this interaction, LTo cells express homeostatic chemokines for B, T and dendritic cells. In the context of atherosclerosis, Grabner et al. discovered that vascular smooth muscle cells (VSMCs) from hypercholesterolemic ApoE KO mice could express CXCL13, CCL21 and CCL19 chemokines in response to a stimulation of their LTβR and could play the role of LTo cells triggering aortic lymphoid neogenesis [4]. We have extended these findings by showing that M1 macrophages, through their secretion of soluble factors, are potent LTi cells conferring a LTo phenotype to VSMCs [9]. In the present work, we aimed at establishing whether these observations are relevant to the human abdominal aortic aneurysm (AAA) disease.

AAAs are evolutive forms of atherothrombosis in humans and interestingly TLOs have recently been detected in their adventitial layer [7]. In addition, surgical repair of AAAs represents a unique opportunity to carry out assays on cells from the adventitia of atherothrombotic arteries. By systematically studying fresh tissue samples deriving from human AAA, we found that AAA’s VSMCs can assume the role of lymphoid tissue organizer cells. We therefore evaluated the putative triggers and functional studies allowed us to show that the transformation of VSMCs into LTo-like cells is guided by AAA micro-environmental cues.

## Materials and Methods

### Human tissue

AAA tissues were obtained from patients undergoing surgery and enrolled in the RESAA protocol (“REflet Sanguin de l’évolutivité des Anévrysmes de l’aorte abdominale”). We used, as control aortas human tissues macroscopically normal and devoid of early atheromatous lesions from deceased organ donors.

### Ethics statement

All patients gave informed written consent, and the protocol for AAAs and control aortas sampling was approved by a French ethics committee (Comité Consultatif de Protection des Personnes dans la Recherche Biomédicale CCPRB Paris-Cochin, approval no 2095). Control aortas were sampled from deceased organ donors with the authorization of the French Biomedicine Agency (PFS 09-007).

### Immunohistochemistry

Human AAAs and control aortas were fixed in paraformaldehyde (PFA) 3.7%. Samples were embedded in paraffin and sectioned at 6 µm. Sections were deparaffinized in toluene, hydrated in ethanol, and incubated in retrieval reagent (R&D) for 20 minutes. After blocking in 5% BSA, slides were incubated with primary antibodies (rabbit anti-human CD3 and mouse anti-human CD20, clone L26, BD Biosciences) overnight at 4°C for extracellular staining. After several washes with PBS, slides were then incubated with the appropriate secondary antibodies (goat anti-mouse and goat anti-rabbit conjugated with either rhodamine or DL649, Jackson Immunoresearch) at RT for 30 minutes.

Intracellular staining for chemokines and α-smooth muscle actin were performed on slides previously fixed and permeabilized with 0.5% Triton and incubated 1 hour at 37°C with primary antibodies (polyclonal goat anti-human CXCL16; goat anti-human CCL20; mouse anti-human α-smooth muscle actin; goat anti-human CXCL13; goat anti-human CCL21; mouse anti-human CCL19, BD Biosciences). Slides were then washed and incubated with secondary antibodies (goat anti-mouse-rhodamine; goat anti-rat-rhodamine; goat anti-rabbit-DL649, Jackson Immunoresearch; donkey anti-goat 546, Invitrogen) for 30 minutes.

Nuclei were stained with Hoechst 53542 and slides were cover-mounted with Prolong Gold Antifade Reagent (Invitrogen). The fluorescence was detected with a Zeiss Axiovert 200 M microscope equipped with the AxioCam MRm vers.3 camera, the ApoTome system and the AxioVision image capture software.

### Human VSMCs and fibroblast primary cultures

The media from human aortic tissues obtained from AAAs or organ donors was microdissected from the adventitia. Tissues were then cut into small fragments, and VSMCs and fibroblasts were separately plated after 3 hours of digestion with 0.22 U/mg collagenase and 4.58 U/mg elastase for the media and 0.22 U/mg collagenase for the adventitia, respectively. Cells were then cultured in Smooth Muscle Cell growth Medium 2 supplemented with 1% PSA, plasmocin, Fetal Calf Serum (FCS), Epidermal Growth Factor, basic Fibroblast Growth Factor, and insulin. At confluence, cells were lysed in Trizol for later RNA extraction. Cytokine stimulation of VSMCs was performed with the indicated concentrations.

### Adventitial inflammatory infiltrate analysis by flow cytometry

Adventitial samples from the aorta of aneurysmal patients or organ donors were cut into small pieces (<1 mm) and incubated in digestion buffer (1 mg/mL Collagenase A and 100 U/mL Dnase1) for 1 hour at 37°C. After filtration and several washing steps, cells were layered on Ficoll-Paque. The lymphoid cell-containing interphase layer was collected and washed twice in PBS. The following antibodies were used to characterize the T and B lymphocyte populations: mouse anti-human CD45-QDot800; mouse anti-human CD3-QDot605 and mouse anti-human CD20-PE-Cy7, BD Biosciences.

Cells were incubated with the antibody mix for 25 minutes at 4°C and analyzed on a LSRII flow cytometer (BD Biosciences). Photomultiplicators for FSC, SSC and fluorescent canals were adjusted on unstained cells. Compensations were established using simple staining realized on compensation beads (CompBeads; BD Biosciences) coupled with antibodies directed against mouse κ chain immunoglobulin. Data were analyzed on DIVA software (BD Biosciences).

### Immunodetection of chemokines and cytokines

Cell culture supernatants from VSMCs were analyzed for CXCL13, CCL19, CCL21, CCL20, CXCL16 and cytokine content using the BioPlex assay (Bio-Rad). Carboxylated beads were coupled with chemokine capture antibodies after activation with N-hydroxysulfosuccinimide (S-NHS) in the presence of (1-ethyl-3-[3-dimethylaminopropyl]) carbodiimide hydrochloride (EDAC). Coupled beads were incubated for 2 hours at room temperature (RT) with VSMC supernatants or recombinant standards of each chemokine. After washing, a biotinylated detection antibody was added to the reaction. A streptavidin-phycoerythrin (streptavidin-PE) reporter complex was then added to reveal the biotinylated detection antibodies. PE fluorescence was analyzed on a Bioplex-200 analyzer (Bio-Rad), and the amount of bound analytes was quantified using standard curves obtained with the recombinant chemokines.

### Preparation of conditioned medium

The media of AAA (n = 20) and control aortas (n = 20) was separated from the adventitia and each layer was cut into small pieces (5 mm^3^). These various tissue samples were then separately incubated (24 hours at 37°C) in a standardized volume (6 mL/g of wet tissue) of RPMI 1640 medium (life technologies France) supplemented with antibiotics and an antimycotic. The conditioned medium thus obtained was centrifuged, and the supernatant was aliquoted and frozen at −80°C until use.

### Chemotaxis experiment

For chemotaxis assays, total peripheral blood mononuclear cells (PBMCs) were isolated from human blood and were added to a polycarbonate filter with 5 µm pores (ChemoTx, Neuro Probe Inc., Gaithersburg, MD, USA) at the concentration of 1.10^6^ cells/well. The filter was gently placed on wells that were filled with human aortic conditioned medium. Following 4 hours of migration at 37°C, cells that had migrated through the filter towards the bottom wells were harvested and stained with mouse anti-human CD3-eFluor 700, mouse anti-human CD4-PE-Cy7, mouse anti-human CD8-APC, mouse anti-human CD45RO-PE-CF594, mouse anti-human CD45RA-V450, and mouse anti-human CD19-AlexiaFluor700, all from BD Biosciences. Cells were then analyzed by flow cytometry, as described above.

### Gene expression analysis

Total RNAs were extracted using the Trizol reagent, and mRNA reverse transcription was performed using the iScript reverse transcriptase (Biorad).

Real time PCR was performed on the CFX 100 (Biorad) cycler using the primers listed in S1 Table. We used 1 ng cDNA of each sample in a total volume of 22 µL that contained forward and reverse primers (250 nM), and the Syber-Green master mix (Biorad). The amplification program was as follows: 1 cycle: 50°C, 2 minutes; 1 cycle: 95°C, 15 minutes; 50 cycles: 95°C 40 sec and 60°C 1 minute. Dissociation curves were analyzed at the end of the amplification, and expression of the genes of interest was normalized by the expression of either hypoxanthine-guanine phosphoribosyltranferase (HPRT) or β-actin, as indicated.

### Statistical analysis

Means, SEM and *p values* were calculated using the software Statview. We considered as statistically significant a probability value <0.05 using Student *t* tests and non-parametric tests (Mann-Whitney), as appropriate.

## Results

### Characterization of lymphoid structures in human AAAs

**Figure 1:**
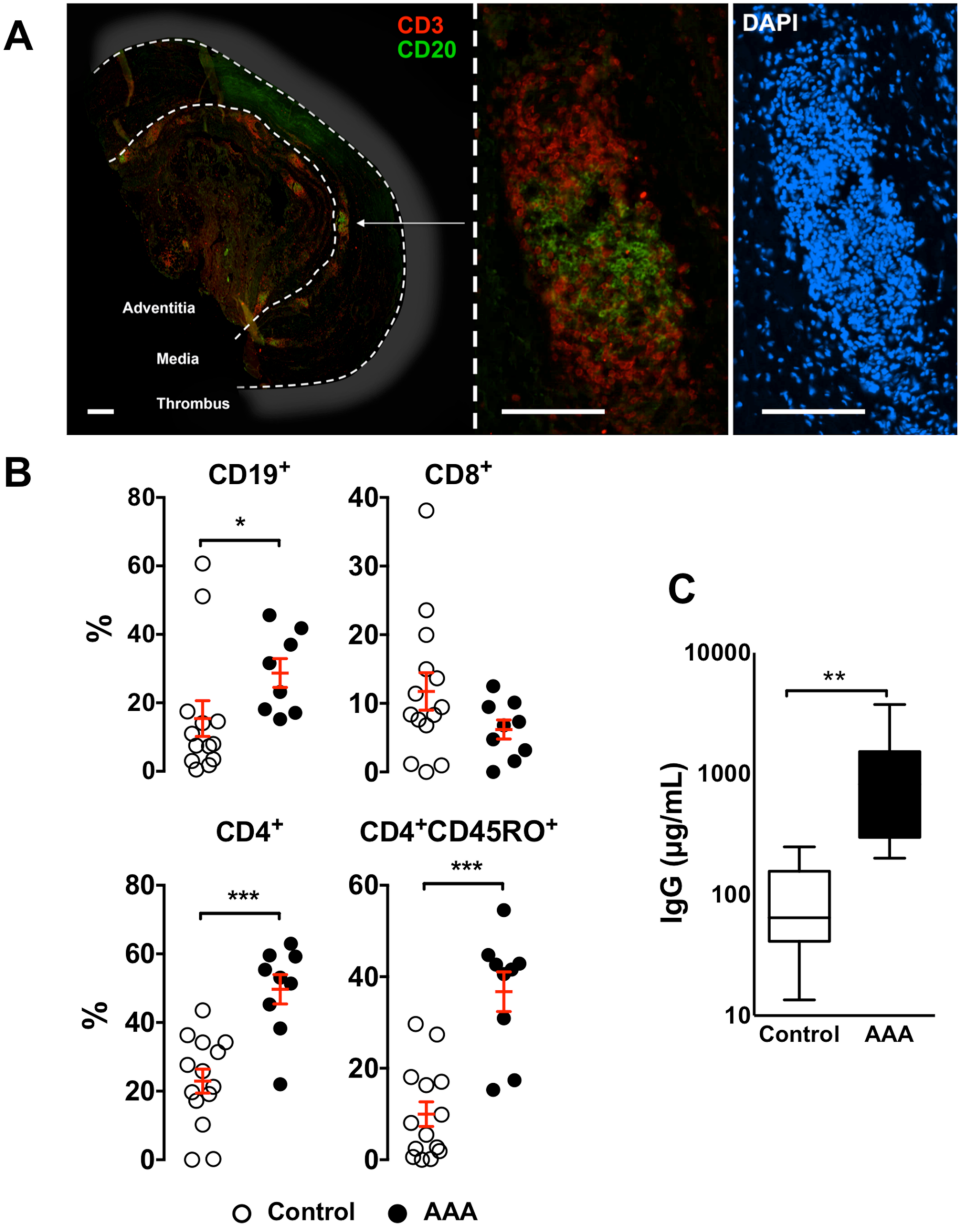
Organized Tertiary Lymphoid Structures in human Abdominal Aortic Aneurysm. (**A**) Representative immunohistochemical staining of CD3^+^ T cells (red), CD20^+^ B cells (green) and nuclei (DAPI, blue) performed on human AAA cross-sections (scale bar = 1 mm). (**B**) Flow cytometry analysis of the percentage of CD19^+^ B cells, total CD8^+^, CD4^+^and memory (CD4^+^CD45RO^+^) T cells in adventitia from AAAs (n = 9) or control aortas (n = 14). (**C**) Box plot showing the total IgG levels in conditioned medium prepared from adventitial tissues of AAAs (n = 14) and control aortas (n = 5) quantified by ELISA. *, p<0.05; **, p<0.01; ***, p<0.001.

In all the AAA tissues analyzed, but none of the control aortas, the immunohistochemical analysis revealed a prototypic organization of the immune infiltrates in the media and the adventitia where massive CD20^+^ B cell follicles were surrounded by T cells (CD3) (Fig. 1A), suggestive of mature aortic TLOs. Flow cytometry was used to establish the phenotype of the leukocytes extravasated in the aorta. Since TLO formation is less frequent and more random in the media, we are providing only the results obtained in the adventitia which are much more robust and reproducible. We found that the percentage of CD19^+^ B cells and CD4^+^ T cells was significantly increased in the adventitia from AAAs compared to control aortas. Furthermore, among the CD4^+^ T lymphocytes, those displaying a memory phenotype (CD4^+^CD45RO^+^) were enriched in AAA adventitia. At variance, the CD8^+^ T cell compartment was similar in the two types of tissues (Fig. 1B). As shown in Fig. 1C, the IgG concentration was significantly increased in the conditioned medium prepared from AAA adventitial tissues as compared to control aortas indicating that the adventitial B cells in AAAs are fully functional and can locally produce immunoglobulins.

### AAA micro-environment is prone to recruit immune cells

**Figure 2:**
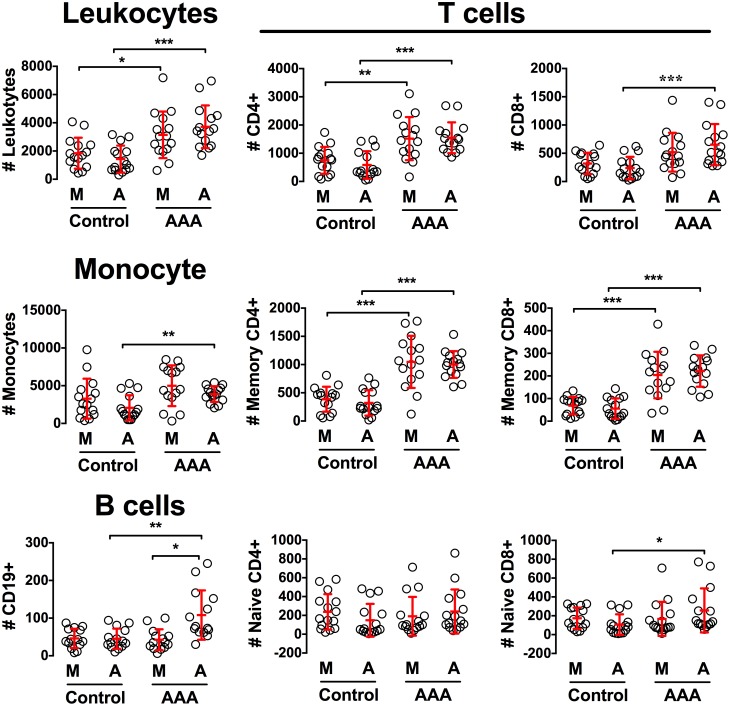
The aneurysmal aortic tissue promotes the chemoattraction of immune cells. Chemotaxis assay on conditioned medium prepared from the media (M) and adventitia (A) of AAAs (n = 15) and control aortas (n = 15). Plotted is the number of total leukocytes, monocytes, CD19^+^ B cells, CD44^+^CD62L^−^ memory CD4^+^ or CD8^+^ T cells, CD44^−^CD62L^+^ naïve CD4^+^ or CD8^+^ T cells that have migrated toward the well containing the conditioned medium. *, p<0.05; **, p<0.01; ***, p<0.001.

The recurrent presence of inflammatory infiltrates in the media and adventitia of human AAAs suggested that these tissues display chemoattractive properties for T and B lymphocytes. In order to test this hypothesis, the adventitial and medial layers from fifteen AAAs and fifteen control aortas were separated by microdissection. Conditioned medium from these split tissues were tested for their ability to trigger the recruitment of T and B cells in a chemotaxis migration assay of blood leukocytes. As shown in Fig. 2, the conditioned medium prepared from the media and adventitia of AAAs recruited significantly more lymphocytes and monocytes than conditioned medium from the respective arterial layers of control aortas. A detailed analysis of the attracted cells showed that the conditioned medium of AAA adventitia was prone to specifically recruit CD19^+^ B cells. In addition, we found that both CD4^+^ and CD8^+^ T cells – and more specifically CD45RA-RO^+^ memory cells – were preferentially chemoattracted by the conditioned medium from the media and adventitia of AAAs.

**Figure 3:**
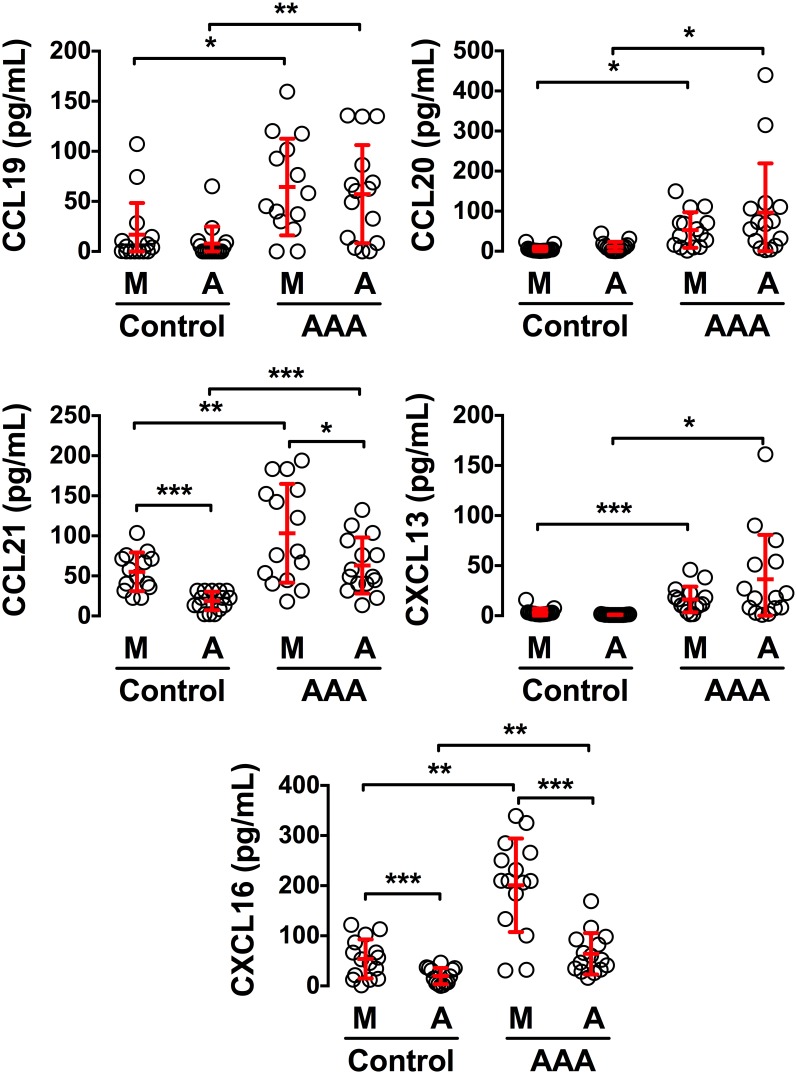
Increased concentration of chemokines in conditioned medium from AAA tissues. The concentration of CCL19, CCL20, CCL21, CXCL13 and CXCL16 was assessed by ELISA assays on conditioned medium prepared from the media (M) and adventitia (A) of AAAs (n = 15) and control aortas (n = 15). *, p<0.05; **, p<0.01; ***, p<0.001.

These results confirm that the media and adventitia within AAA are releasing chemoattractive molecules able to trigger the recruitment of immune cells. We therefore next analyzed the concentration of various chemokines in the conditioned medium prepared from the split arterial layers. Remarkably, we found in conditioned medium from AAA tissues significantly higher levels of CCL19, CCL20, CCL21, CXCL13 and CXCL16 chemokines as compared to control aortas (Fig. 3). Moreover, this analysis revealed distinct chemokine concentrations between the media and the adventitia. Indeed, the media contained significantly more CCL21 and CXCL16 than the adventitia, both in AAA and controls, while CCL19, CCL20 and CXCL13 were similar in the two layers. Taken together, these results demonstrate that AAA tissues produce chemokines at levels that are biologically relevant since they are sufficient to trigger the recruitment of T and B cells, thereby participating to the formation of aortic TLOs. At these late stages of the disease, this chemokine production can be attributed either to the stromal cells that reside in the vascular wall and/or to the immune cells themselves that constitute the TLOs. We therefore next to evaluated whether human vascular stromal cells contribute to the formation of aortic TLOs through their secretion of chemokines as shown for mouse vessels [4], [9], [10].

### Aortic smooth muscle cells behave like organizer stromal cells in AAA

**Figure 4:**
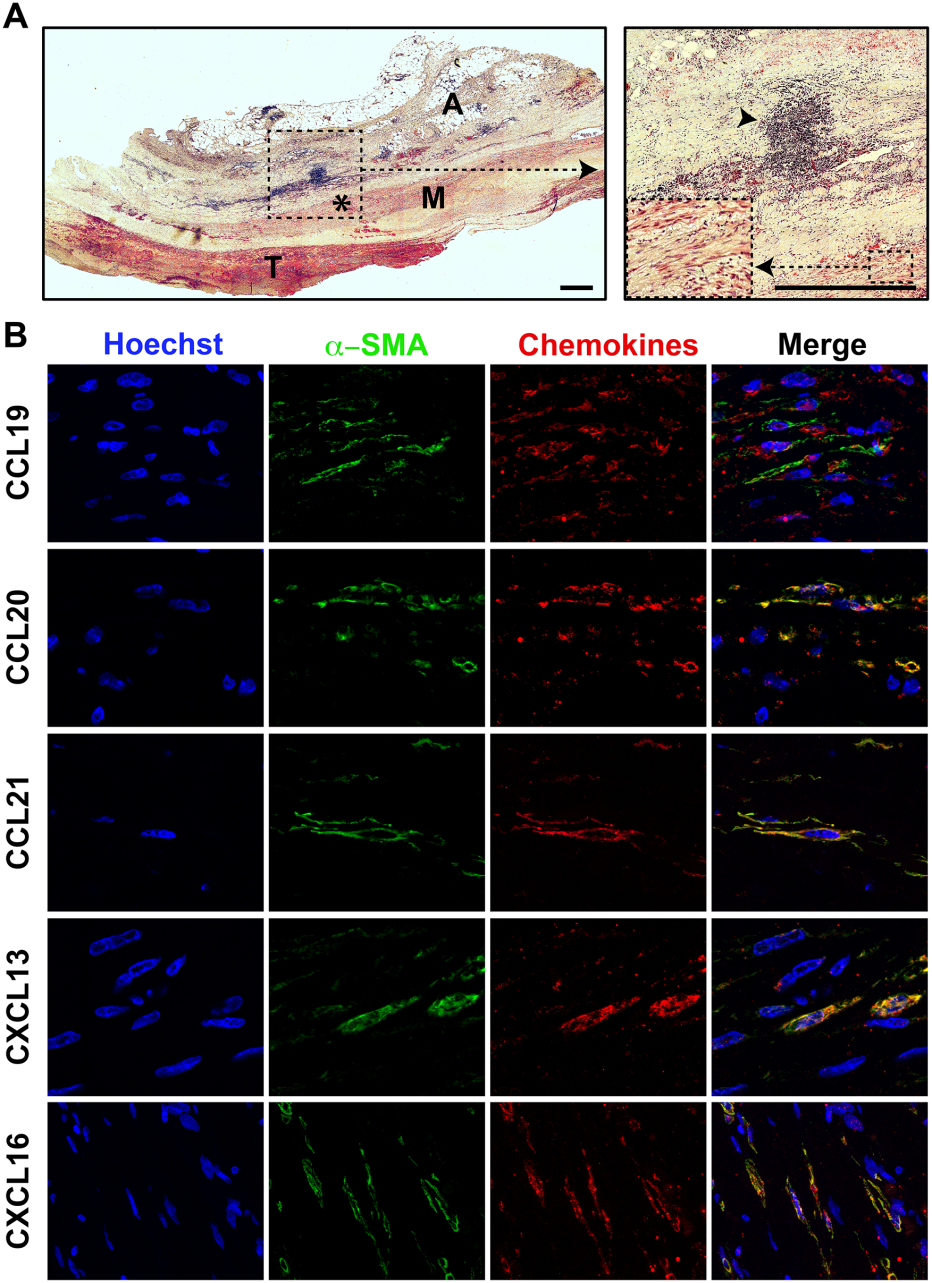
VSMCs in the vicinity of human aortic TLOs produce chemokines. (**A**) Masson’s trichrome staining performed on a human AAA cross-section. The dashed square indicates the area that is magnified on the right inset. The asterisk represents the smooth muscle cell-rich area that is magnified in the dashed square box in the right inset (M = media; A = adventitia; SLO = Secondary Lymphoid Organ; scale bar = 2 mm). (**B**) Representative immunohistochemical staining of α-SMA^+^ VSMCs (green), CCL19, CCL20, CCL21, CXCL13 and CXCL16 chemokines (red) and nuclei (DAPI, blue) performed on human AAA cryosections (scale bar = 50 µm; n = 5).

**Figure 5:**
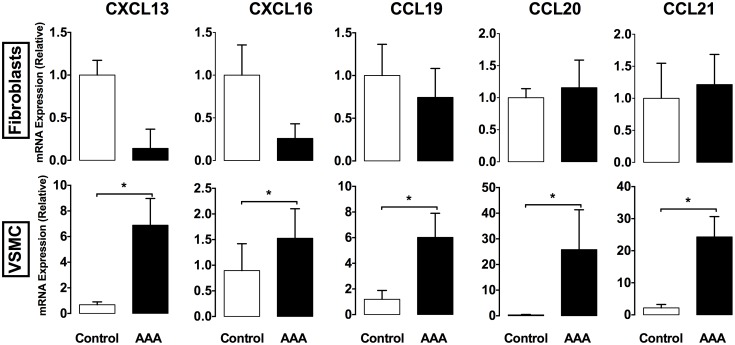
VSMCs, but not fibroblasts, from AAA patients express chemokines. (**A**) The expression of CCL19, CCL20, CCL21, CXCL13 and CXCL16 chemokines was determined by RT-qPCR on RNA extracted from primary cultures of adventitial fibroblasts and medial VSMCs from AAA and healthy subjects. Data were analyzed using the 2^−ΔΔCt^ Pfaffl formula [30] in which data from AAAs were compared to Ct from control aortas, normalized to the Ct values of the β-actin. Data are representative of 4 independent experiments. *, p<0.05.

The production of homeostatic chemokines was explored by immunohistochemistry on human AAA aortic cross sections. As shown in Fig. 4, we found VSMCs in the media of AAAs that highly expressed CCL19, CCL20, CCL21, CXCL13 and CXCL16 chemokines. At variance, we failed to detect adventitial fibroblasts positive for any chemokine (data not shown). These data indicate that VSMCs in human AAAs behave, as reported in ApoE KO mice [4], [9], [10], like lymphoid tissue organizer (LTo) cells. The RT-qPCR analyses performed on mRNAs extracted from primary cultures of medial VSMCs and adventitial fibroblasts from AAAs and control aortas confirmed these results. Indeed, we found that transcripts for CXCL13, CXCL16, CCL19, CCL20 and CCL21 were all significantly increased in VSMCs from AAA patients while no difference was found between adventitial fibroblasts from AAAs and control aortas (Fig. 5).

Interestingly, we also found that the overexpression of these chemokines was not maintained upon passages of VSMC primary cultures (data not shown), ruling out an epigenetic regulation of their production and instead suggesting that the variations observed were rather due to the persistent presence of inducing micro-environment cues in atherothrombotic arteries.

During embryonic lymphoid organogenesis, the stromal cells are converted into LTo cells by CD45^+^CD4^+^CD3^−^ LTi cells that create a “local physiological” inflammation through the expression of TNF-α and lymphotoxins. We could not find any CD45^+^CD4^+^CD3^−^ prototypic LTi cells in the inflamed arterial wall (data not shown). In the absence of LTi cells, we hypothesized that inflammatory molecules produced in the aneurysmal tissue could confer the chemokine-expressing profile to VSMCs in the media. We focused on a large panel of proinflammatory cytokines that have been found to be associated to atherothrombotic disease.

### Soluble inflammatory mediators concentrate in AAA tissues and drive the conversion of aortic VSMCs into LTo

**Figure 6:**
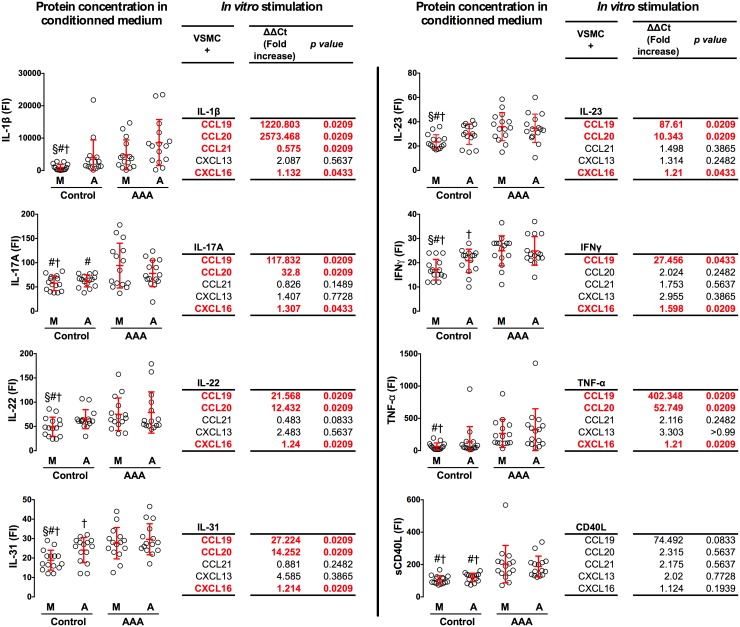
Inflammatory chemokines produced in AAA tissues trigger the expression of chemokines by VSMC. The graphs represent the relative concentrations of IL-1β, IL-17A, IL-22, IL-31, IL-23, IFNγ, TNF-α and CD40L that were assessed by ELISA assays on conditioned medium prepared from the media (M) and adventitia (A) of AAAs (n = 15) and control aortas (n = 15; §, #, †, p<0.05 vs Control A, AAA M and AAA A, respectively). The tables represent the expression of CCL19, CCL20, CCL21, CXCL13, and CXCL16 chemokines by human VSMCs that were stimulated for 15 hours with rIL-1β (5 ng/mL), rIL-17A (50 ng/mL), rIL-22 (10 ng/mL), rIL-31 (50 ng/mL), rIL-23 (25 ng/mL), rIFNγ (10 ng/mL), rTNF-α (1 ng/mL), and rCD40L (100 ng/mL). These cytokine concentrations were 2 log higher than those detected in the conditioned medium, so as to compensate for the *in vivo* situation where cells are subjected to chronic, additive and combined stimulations. The relative expression was determined by RT-qPCR on extracted RNA, and data were analyzed using the 2^−ΔΔCt^ Pfaffl formula [30] in which Ct values from stimulated VSMCs were compared to unstimulated cells, and normalized to the Ct values of β-actin. Data are representative of 4 independent experiments.

The concentrations of IL-1β, IL-17A/F, IL-22, IL-23, IL-31, IFNγ, sCD40L and TNF-α were analyzed in the conditioned medium prepared from AAAs or control aortas. Our results show that IL-1β, IL-17A, IL-22, IL-23, IL-31, sCD40L, IFNγ and TNF-α were all significantly increased in the conditioned medium from aneurysmal aortic tissues as compared with control tissues (Fig. 6; dotplots). We then tested individually these molecules in order to evaluate whether any of them could elicit the expression of CCL19, CCL20, CCL21, CXCL13 and CXCL16 chemokines by primary cultured human VSMCs. We found that IL-1β, IL-17A, IL-22, IL-23, and TNF-α were able to consistently induce the transcription of both CCL19 and CCL20 by VSMCs whereas IFNγ was effective solely on the expression of CCL19 (Fig. 6 Tables). The effect of these recombinant cytokines drove also a slight but significant increase in the expression of CXCL16 by primary VSMCs. Although none of the tested cytokines could induce a detectable effect on the expression of CXCL13 and CCL21, these findings strongly suggest that the inflammatory environment in AAA tissues is able to drive the phenotypic changes of VSMCs towards chemokine-expressing LTo-like cells.

## Discussion

It is now widely established that the formation of ectopic lymphoid structures – which resemble the SLOs, in terms of function and cell composition – can occur in any tissue subjected to a persistent and chronic inflammation. Those circumstances are encountered in a large panel of diseases, such as cancers, autoimmune, and infectious disorders [11]–[14]. In the context of atherosclerosis, TLOs were detected in the adventitia of human atherothrombotic arteries as early as in the 1950’s [15], [16]. These peri-arterial TLOs have been further investigated in the last decade, both in human and mouse arteries, leading to a better knowledge of their structure and cell content. Herein, we describe the presence of lymphoid aggregates located in the adventitia and in the external part of the media of abdominal aortas with advanced aneurysms. These lymphoid nodules are defined as TLOs since they are composed of B cell clusters surrounded by T cells, a prototypic organization reported for ectopic germinal centers. In the hypercholesterolemic ApoE KO mouse, we [17] and others [4], [10] have demonstrated that, as it the case for the development of SLOs, the formation of TLOs requires, at the first place, the interaction between a LTi cell and a stromal cell that confers to the stromal cell the ability to behave as a LTo cell i.e. the capacity to produce a panel of homeostatic chemokines. The fact that mouse VSMCs were shown to express CCL19, CCL20, CCL21, CXCL13, and CXCL16 chemokines is a strong indication that these cells could take the role of lymphoid tissue organizer cells in atherosclerotic vessels [4], [9], [10]. The present report demonstrates that human VSMCs can also act as LTo cells and could thereby be involved in human aortic lymphoid neogenesis.

AAA is a clinical situation during which a non-occluding luminal thrombus is constituted on atherosclerotic lesions. Surgery is recommended when the aneurysm is large enough (>5.5 cm in diameter) and that the risk of surgery is less than the risk of rupture. In open surgery, the surgeon opens the abdomen and stitches in a replacement section of artery. This surgery is one of the rare case for which we have access to fresh human medial and adventitial tissues.

In particular, we could run flow cytometry analysis on digested AAA tissues, which revealed the presence of B cells in the adventitial layer with a very high prevalence. This observation prompted us to perform a more comprehensive study in this clinical setting and we found that 100% of aneurysms displayed significant B and T cell infiltrates organized into TLOs indicating that this pathological condition is particularly propitious for TLO formation and represents therefore an appropriate clinical setting to further study the mechanisms leading to the adventitial lymphoid neogenesis.

In support of this, we found that AAA tissues were more prone for leukocyte recruitment than non-AAA tissue samples. These observations were strongly corroborated with the high amount of chemokines detected in AAA tissues, while they could be barely spotted in non-AAA tissues.

Aortic TLOs are located in the vicinity of the two main stromal cells of the artery: the VSMCs and the adventitial fibroblasts. We found that medial VSMCs, but not adventitial fibroblasts, from AAA patients expressed dramatically more CCL19, CCL20, CCL21, CXCL13, and CXCL16 chemokines than cells from control aortas. These findings strongly suggest that VSMCs, as shown for mouse tissues [4], [10], [17], play the role of LTo cells in human AAAs.

We next aimed at identifying the cellular and molecular factors conferring VSMCs with their LTo potential. During organogenesis of lymph nodes, this role is taken by CD45^+^CD4^+^CD3^−^ LTi cells. We could not detect any cell displaying this phenotype in the aortas that we have analyzed and to the best of our knowledge, such cells have never been detected in inflamed aortas or in any inflammatory condition associated with TLO formation. Furthermore, we have previously shown that, in a murine model of atherosclerosis, soluble inflammatory molecules were able by themselves to trigger lymphoid neogenesis in the aortic wall [17].

We found that conditioned medium prepared from AAA tissues contained high levels of several inflammatory soluble cytokines. In particular, we found high levels of IL-1β, TNF-α, IFNγ and CD40L, all of which have been associated with the progression of atherosclerosis [18]–[21]. Also, an increasing body of evidence points at the contribution of the Th17 immune response to the disease [22] and our results indicate that AAA tissues can release significantly more IL-17, IL-22, and IL-23, the key Th17-interleukin triad. So was the case for IL-31, an interleukin specifically expressed by memory CD4 T cells [23], an observation that fits with the results in the *in vitro* chemotaxis assay where memory CD4 T cells were preferentially recruited by the conditioned medium from AAA tissues.

We then directly assessed whether each of these cytokines could induce the expression of chemokines by VSMC in an *in vitro* assay. Among all the cytokines analyzed, only the CD40L was unable to trigger the expression of chemokines by VSMCs. The effect of IL-1β and TNF-α stimulation on chemokine expression was the most robust one with an increase of the expression of CCL19, CCL20, and CXCL16, as described on other cell types [10], [24]–[26]. IFNγ was able to trigger the expression of CCL19 and CXCL16 by VSMCs, in agreement with previous reports [25], [27]. As reported [28], [29], we found that IL-17A and IL-23, but also IL-22 and IL-31, induced the expression of CCL20. These four interleukins triggered the expression of CCL19 and CXCL16 by VSMCs. Surprisingly, none of these cytokines had an impact on the expression of CXCL13 and CCL21, which are two major chemokines involved in the formation of SLOs, indicating that other inducing molecules are still to be characterized in the context of atherothrombotic arteries.

All these findings suggest that in human atherothrombotic arteries, local inflammation activates VSMCs and confers them the phenotype of LTo cells that express CCL19, CCL20, and CXCL16 chemokines. This participates to the recruitment of immune effectors and may thereby critically contribute to the formation of aortic TLOs.

## Supporting Information

S1 Table Primer list.
(TIF)

S2 Table VSMC express chemokines in response to inflammatory cytokines. Human VSMCs were stimulated for 15 hours with rIL-1β (5 ng/mL), rIL-17A (50 ng/mL), rIL-22 (10 ng/mL), rIL-31 (50 ng/mL), rIL-23 (25 ng/mL), rIFNγ (10 ng/mL), rTNF-α (1 ng/mL), and rCD40L (100 ng/mL), and the relative expression of CCL19, CCL20, CCL21, CXCL13, and CXCL16 chemokines was determined by RT-qPCR on extracted RNA. Data were analyzed using the 2^−ΔΔCt^ Pfaffl formula [30] in which Ct values from stimulated VSMCs were compared to unstimulated cells, and normalized to the Ct values of β-actin. Data are representative of 4 independent experiments.
(JPG)
