# Supplementary figures and images for: Inflammatory Micro-Environmental Cues of Human Atherothrombotic Arteries Confer to Vascular Smooth Muscle Cells the Capacity to Trigger Lymphoid Neogenesis
Source: PLoS One. 2014 Dec 30;9(12):e116295. doi: 10.1371/journal.pone.0116295 (PMC4280229; doi:10.1371/journal.pone.0116295)

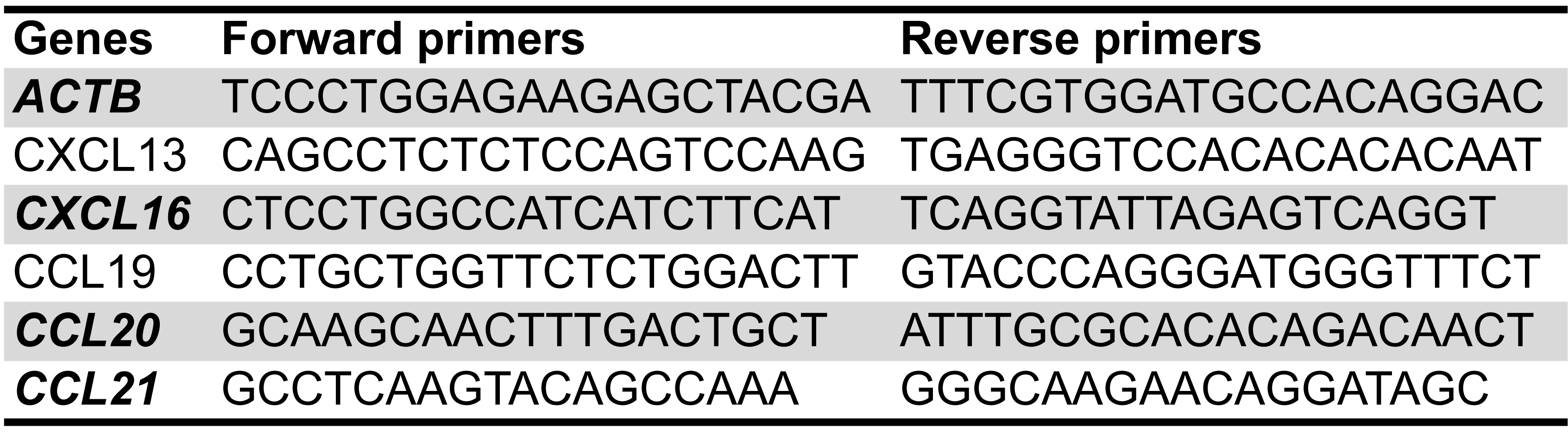

Supplement: S1 Table — Primer list. (TIF) [file pone.0116295.s001.tif]

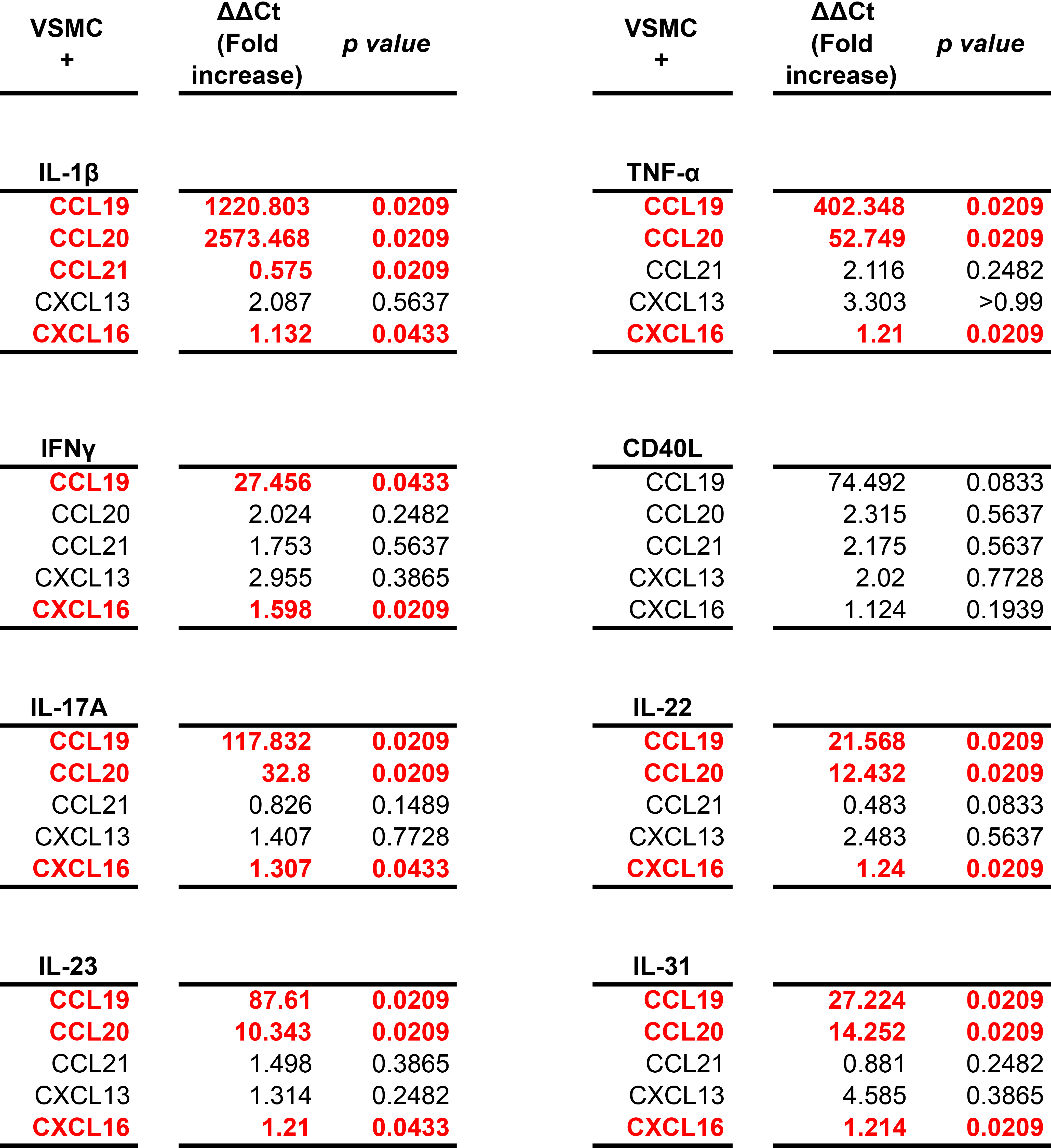

Supplement: S2 Table — VSMC express chemokines in response to inflammatory cytokines. Human VSMCs were stimulated for 15 hours with rIL-1β (5 ng/mL), rIL-17A (50 ng/mL), rIL-22 (10 ng/mL), rIL-31 (50 ng/mL), rIL-23 (25 ng/mL), rIFNγ (10 ng/mL), rTNF-α (1 ng/mL), and rCD40L (100 ng/mL), and the relative expression of CCL19, CCL20, CCL21, CXCL13, and CXCL16 chemokines was determined by RT-qPCR on extracted RNA. Data were analyzed using the 2−ΔΔCt Pfaffl formula [30] in which Ct values from stimulated VSMCs were compared to unstimulated cells, and normalized to the Ct values of β-actin. Data are representative of 4 independent experiments. (JPG) [file pone.0116295.s002.jpg]
